# Context-Aware and Locality-Constrained Coding for Image Categorization

**DOI:** 10.1155/2014/632871

**Published:** 2014-03-18

**Authors:** Wenhua Xiao, Bin Wang, Yu Liu, Weidong Bao, Maojun Zhang

**Affiliations:** College of Information System and Management, National University of Defense Technology, Changsha, Hunan 410073, China

## Abstract

Improving the coding strategy for BOF (Bag-of-Features) based feature design has drawn increasing attention in recent image categorization works. However, the ambiguity in coding procedure still impedes its further development. In this paper, we introduce a context-aware and locality-constrained Coding (CALC) approach with context information for describing objects in a discriminative way. It is generally achieved by learning a word-to-word cooccurrence prior to imposing context information over locality-constrained coding. Firstly, the local context of each category is evaluated by learning a word-to-word cooccurrence matrix representing the spatial distribution of local features in neighbor region. Then, the learned cooccurrence matrix is used for measuring the context distance between local features and code words. Finally, a coding strategy simultaneously considers locality in feature space and context space, while introducing the weight of feature is proposed. This novel coding strategy not only semantically preserves the information in coding, but also has the ability to alleviate the noise distortion of each class. Extensive experiments on several available datasets (Scene-15, Caltech101, and Caltech256) are conducted to validate the superiority of our algorithm by comparing it with baselines and recent published methods. Experimental results show that our method significantly improves the performance of baselines and achieves comparable and even better performance with the state of the arts.

## 1. Introduction 

Automatic image categorization has drawn increasing attention of the researchers around the world due to its widespread prospects in various applications (e.g., video surveillance [1], image and video retrieval [2], web content analysis [3], and biometrics [4]). In recent works addressing the image categorization tasks, the BOF based model [5], developed from the BOW (Bag-of-Words) in document analysis [6], is one of the most popular and efficient models in dealing with this problem. BOF based method is often comprised of the following common steps: feature extraction, codebook (or dictionary) designing, feature encoding, and pooling. Given a dataset, firstly, local features are often depicted by descriptors such as SIFT [7]. Secondly, a codebook to span the feature space is often designed by *K*-means [8], sparse coding [9], K-SVD [10], and others. Thirdly, given feature descriptors and codebook as the input, the output of this step is a coding matrix. In this step, each feature descriptor activates a number of code words and generates a coding vector after features are coded over this codebook. Fourthly, pooling methods (e.g., average pooling [8] and max pooling [11]) are often used to obtain the compact signature of the image. Of all the above four steps, feature coding is the core component, which links feature extraction and feature pooling, and greatly influences image classification in terms of both accuracy and speed [12]. Owning to this key role of coding phase in the pipeline of BOF based method, since the seminal work of [8], improving the coding strategy has drawn increasing attention in recent works.

**Figure 1:**
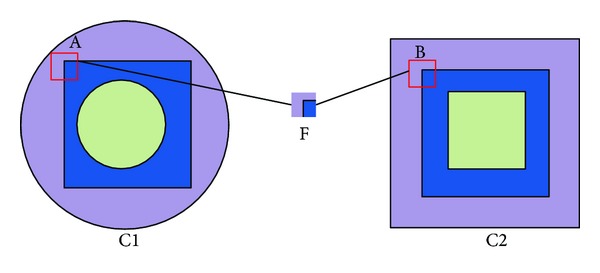
Two ambiguous features A and B are extracted from two different image classes and described with a similar descriptor F.

Coding can be regarded as a procedure assigning few code words with weighted coefficient to represent local features while satisfying some desirable properties. Various coding styles have been proposed in previous literatures [8, 10, 11, 13–17]. And some limitations (e.g., quantization error, nonconsistency, and computational cost) of traditional models have been partially alleviated by those previous works. However, there still* exists an important limitation of BOF* that cannot be solved by previous works.* This limitation is produced* by the features from different classes to depict different objects while with similar descriptor. Intuitively, those features should be encoded discriminatively as to preserve their different semantic meaning. However, due to their similar descriptors, they cannot be distinguished with their codes generated by previous coding strategies. This is named* ambiguity problem*. For example, as shown in Figure 1, there are two ambiguous features A and B. A and B, with different semantic meaning, indicate corner patch in two images, respectively. Obviously, A and B should be encoded with different bases to distinguish the two images better. However, due to the reason that they have similar descriptors, they are given similar codes and cannot be distinguished clearly by previous coding strategies such as VQ, SVQ, SC, and LLC.

In this paper, we attempt to further improve the BOF with regard to the above-presented ambiguity problem. The* motivations* of our method are as follows. (1) Scene or object in realistic image has a certain cooccurrence pattern, which determines the difference of each class images, around its neighborhood. So we can use the cooccurrence pattern (context) information to distinguish the ambiguous features to solve the ambiguity problem. (2) Inspired by LLC which enforces locality in feature space and achieves excellent performance, we incorporate the locality in context space into LLC so as to inherit its advantages (analytical solution and real-time coding speed).

In detail, observing the realistic images, it can be easily obtained that each class of scene or object has a certain cooccurrence pattern in its neighborhood. For example, a pan and a stove often appear in their neighborhood in kitchen, and a butt often appears near to a barrel in AK47. This appearance cooccurrence can be considered as the context preserving discriminative information of each class. Even though those descriptors from different classes are similar, their context often appears different because their surroundings often show different appearance cooccurrence pattern. Based on above assumption, if we consider the context information when encoding the feature, the coding result of those similar features with different semantic meaning will be discriminative. Therefore, in this paper, we propose to use this context information to tackle the ambiguity problem. Obviously, how to describe such context information and incorporate it into coding procedure become the main tasks of our approach. For this purpose, firstly, to capture the contextual information, a word-to-word co-occurrence relationship matrix for each class is constructed within local domain of each image. Because the statistical relationship matrix reflects the spatial distribution and the cooccurrence of features in neighbor region of each class, it has the ability to describe partial contextual information. Secondly, the relationship matrix is used to select the optimal bases in context space. Thirdly, combined with the locality factor in feature space, this context factor enhances the LLC [15] model to a novel model called context-aware and locality-constrained coding (CALC). Indeed, CALC can be considered a fineness version of LLC because it locally constrains the coding in both feature space and context space. Here, the expression “*context*” means the surrounding appearance cooccurrence pattern of a local feature. Extensive experiment demonstrated the effectiveness of the proposed method.

The rest of the paper is organized as follows.  Section 2 reviews the related feature coding methods. CALC is proposed in Section 3. The details of implementation of CALC are introduced in Section 4. Properties analysis of CALC is presented in Section 5. Then, the experimental results and analysis are shown in Section 6. Finally, conclusion is drawn in Section 7.

## 2. Related Works

BOF based models are widespread adopted in computer vision and pattern recognition fields. In this section, we concentrate on those related works in the view of image categorization here. Let **X** = {*x*_*i*_ ∈ *R* ^*D*^, *i* ∈ 1,…, *N*} be *N* local descriptors with *D*-dimension extracted from an image. Given a codebook with *M* bases **B** = [**b**_1_, **b**_2_,…, **b**_*M*_] ∈ **R** ^*D*×*M*^, **x**_*i*_ is converted into *M*-dimensional code denoted as *c*_*i*_ ∈ **R** ^*M*^ by feature coding methods. Several popular coding methods are as follows.

*Vector Quantization (VQ) [8, 19]*. In the original BOF model, its coding strategy assigns just a single base to the feature, which is known as VQ (Vector Quantization), or HC (Hard Coding). Each local descriptor is assigned to the nearest visual word:

(1) cij={1,if  j=argminj||xi−bj||22,0,else.

This coding is simple but, as reported in [11], suffers from the reconstruction error due to the reason that it only assigns a single code word to the descriptor.

*Soft Vector Quantization (SVQ)*. To ameliorate the quantization loss of VQ, Gemert et al. [13] proposed SVQ on which a feature is coded across many codebook elements instead of using one:

(2) cij=exp⁡(−β||xi−bj||22)∑l=1Mexp⁡(−β||xi−bl||22),

where *β* is a parameter controlling how widely the assignment distributes the weight across all the code words. A small *β* gives a broad distribution, while a large *β* gives a peaked distribution, more closely approximating hard assignment. This is further improved by Liu et al. [14], who use localized soft assignment (LSVQ). Their difference is that SVQ encodes the descriptors across all the codebook elements while LSVQ confines the soft assignment to a local neighborhood around the descriptor being coded.

*Sparse Coding (SC) [11]*. Another way to alleviate the quantization loss of VQ is SC which encodes a descriptor by using the coefficients of a linear combination of the code words in **B**, with a sparsity-promoting *l*_1_ norm:

(3) ci=argminc||xi−Bc||22+λ||c||1, λ∈R,

where the first term represents the reconstruction error of **x**_*i*_ with respect to codebook **B**. The second term is a sparse constraint regularization on code **c**, and *λ* is a regularization factor to balance these terms. Although SC significantly improved its robustness to the problems produced by VQ, its expensive computational demanding and nonconsistent encoding of similar descriptors are the limitations [15].

*Locality-Constrained Linear Coding (LLC) [15]*. To alleviate the limitations of SC, LLC enforces locality instead of sparsity. LLC uses the following criteria:

(4) ci=argminc||xi−Bc||22+λ||d⊙c||22s.t. 1Tc=1,

where the first term is reconstruction error. The second term is the locality constraint regularization on code **c**, and *λ* is a regularization factor. In the second term, ⊙ denotes the element-wise multiplication, and **d** ∈ *R* ^*M*^ is the locality adaptor that gives different weight for each base vector proportional to its similarity to the input feature **x**. Specifically, **d** = exp⁡(dist⁡(**x**_*i*_, **B**)/*σ*), where dist⁡(**x**_*i*_, **B**) = [dist⁡(**x**_*i*_, **b**_1_),…, dist⁡(**x**_*i*_, **b**_*M*_)]^*T*^, and **d** **i** **s** **t**(**x**_*i*_, **b**_*j*_) is the Euclidean distance between **x**_*i*_ and the *j*th base **b**_*j*_. *σ* is used for adjusting the weight decay speed for the locality adaptor. This coding style is based on the hypothesis that descriptors approximately reside on a lower dimensional manifold in an ambient descriptor space; thus, it alleviates the quantization error while preserving the consistent encoding ability.

*Laplacian Sparse Coding (LSC) [16]*. Another alternative approach to improve the consistency of SC is LSC coding strategy, which adds a Laplacian matrix to the SC object function and codes all the descriptors simultaneously:

(5) argminB,C||X−BC||22+λ∑i||ci||1+βtr⁡(CLCT)s.t. ||bj||2≤1, ∀j,

where **L** = **A** − **W** is the Laplacian matrix obtained from the similarity matrix **W** encoding the relationship between local features and **A**_*m*,*m*_ = ∑_*n*_ **W**_*m*,*n*_. By incorporating the similarity preserving term into the objective of sparse coding, Laplacian sparse coding can alleviate the instability of sparse codes. However, since the Laplacian matrix often has an extremely high dimension, LSC is computationally infeasible.

*Locality-Constrained and Spatially Regularized Coding (LCSR) [17].* A novel coding strategy called LCSR is proposed most recently; unlike the previous works, this approach introduces the spatial information into the coding process and its object function leads to the following optimal assignment configuration:

(6) argmin∑p∑i=1m||xp−b^p,i||22+β∑p~qwp,q∑i=1m||b^p,i−b^q,i||22,

where ${\hat{B}}_{p}=\{b_{p,i};i=1,\ldots ,m\}$ denotes the set of code words in **B** assigned to the local feature **x**_*p*_, *p* ~ *q* indicates the indexes of the spatially neighboring patches under a fixed neighboring system, and *w*_*p*,*q*_ is a local regularization parameter that corresponds to the similarity between local patches **x**_*p*_ and **x**_*q*_; the more similar the local patches are, the higher the basis selection operation is regularized. *β* controls the global regularization. Indeed, this assignment style aims at assigning features to bases of cardinality *m* within the set of the *k*-nearest visual words in the codebook while preserving the consistency of the coding regarding the context of the image. Once each local feature is assigned to the optimal bases by solving (6), its response over the selected bases can be obtained using several recent coding strategies (e.g., VQ, SVQ, and LLC). Since it enforces the locality in both the feature space and the spatial domain of the image, as reported in [17], LCSR improves the performance of most of the previous coding schemes when it is integrated into them. However, the object function in (6) is nonconvex and the *α*-expansion based optimization algorithm is adopted, which lead to computational iteration to get the convergence to a local optimum.

All the aforementioned coding schemes overcome some of the limitations of BOF mentioned in Section 1, and from which we can illustrate the comparison between those coding styles in various aspects. As can be seen in Table 1, none of the coding styles has considered the ambiguous coding problem. In the next section, we propose an efficient and effective method to solve this problem.

## 3. Proposed Method

The main components of our method consist of two steps: constructing the word-to-word cooccurrence matrix to describe the local spatial context information and incorporating this context information into coding step. The details of these two aspects are presented as follows.

### 3.1. Construction of Word-to-Word Cooccurrence Matrix

**Figure 2:**
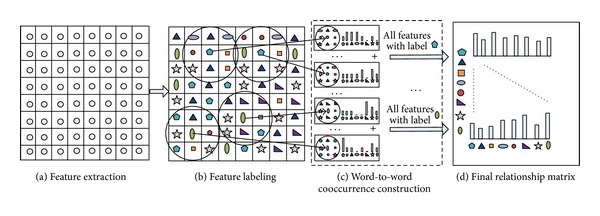
The flowchart of the construction procedure of word-to-word cooccurrence matrix. (a) Extracting features using sift descriptor. (b) Assigning labels to the features using *k*-means. (c) Constructing word-to-word cooccurrence of each visual word. (d) Concatenating the cooccurrences of all visual words to form the final matrix.

As mentioned in Section 1, in a specific scene or object, images often share a common or similar cooccurrence pattern in local region. On the level of descriptor, we believe that such pattern was reflected by the cooccurrence relations among the local descriptors in local region of images. In this section, we present a novel and simple way to describe this relationship, the flowchart of the procedure for one image is illustrated in Figure 2, and the final matrix of the specific class is obtained by accumulating the result of each image belonging to this class. The details of the procedure (for one image) are presented as follows.

With the training data from all classes (e.g., randomly selected 100,000 descriptors from whole datasets), a codebook with size *K* is firstly built by codebook training method (e.g., *k*-means or SC). For a specific image class, let the local descriptors **X** = {*x*_*i*_ ∈ *R* ^*D*^, *i* ∈ 1,…, *N*_*t*_} from this class be training data; then, the local descriptors are labeled using k-NN. We denote *f*_*i*_ = {*x*_*i*_, *l*_*i*_, *p*_*i*_} as the *i*th local feature, where *x*_*i*_ is the descriptor, *l*_*i*_ (belonging to 1,…, *K*) is the corresponding index of code words, and *p*_*i*_ = {*x*_*i*_, *y*_*i*_} records the pixel location at which feature *f*_*i*_ is centered. Thus, all of features can be clustered into *K* sets denoted as *S* = {*S*_1_,…, *S*_*K*_}, where *S*_*i*_ = {*f*_1_^*i*^,…, *f*_*N*_*i*__^*i*^} contains the features with the label of *i* and *N*_*i*_ is the number of features in *S*_*i*_.

**Figure 3:**
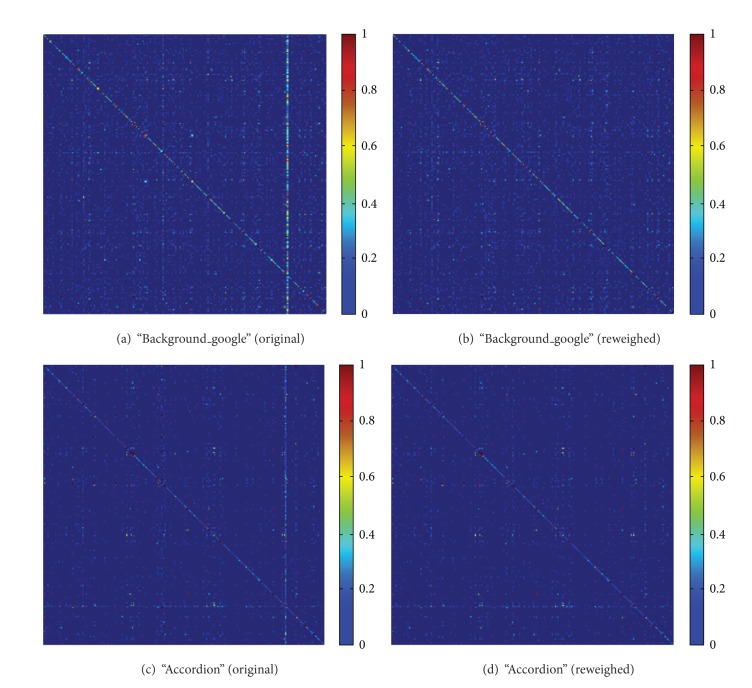
The relationship matrixes presented in depth map. “Original” represents the matrix before reweighing, and “reweighed” represents the matrix after reweighing. As can be seen, the lightest column of the original matrix disappeared after reweighing. It is best to magnify this figure for comparison.

To capture the relations among local features, we define the context domain of feature *f*_*i*_ as follows:

(7) Ci={fj ∣ {xj,yj}∈Ω(pi)},

where *Ω*(*p*_*i*_) denotes the local domain of feature *f*_*i*_, which is represented by a circle with the center of *p*_*i*_ and the radium of *r* (as shown in Figure 2). Thus, the context domain of *f*_*i*_ contains all features within the boundary of the local area *Ω*(*p*_*i*_). Then, for the *j*th feature *S*_*ij*_ in *S*_*i*_, a *k*-dimensional vector *h*_*ij*_ = [*v*_1_^*ij*^,…, *v*_*K*_^*ij*^] is obtained within context domain *C*_*i*_, where *v*_*l*_^*ij*^  (*l* = 1,…, *K*) is the number of features with the label of *l* within the context domain of *S*_*ij*_. After accumulating the vectors of all features in *S*_*i*_, a neighbor distribution histogram *h*_*i*_ of the *i*th code word is obtained:

(8) hi=∑j=1Nihij,

where *h*_*i*_ = [*v*_1_^*i*^,…, *v*_*K*_^*i*^] and *v*_*k*_^*i*^ describe the cooccurrence intensity between the *i*th code word and the *k*th code word. If we denote the code words as vertexes and their cooccurrence intensity as their connection weight, this relationship can be shown as a relationship graph. In this paper, we regularized the value of the connection weight to [0,1]. Once we repeat the above procedure over all *S*_*i*_, *i* = 1,…, *K*, a relationship matrix of all the code words is constructed. We denote it as *H* = [*h*_1_; *h*_2_ …; *h*_*K*_]. For distinguishing the relation matrix constructed on test images, we call this matrix generated by trained data as template matrix. As can be seen in Figures 3(a) and 3(c), two distinguished relationship matrixes constructed from category “Background_google” and “accordion” in Caltech101 are illustrated as depth map, which shows the difference of local context between “Background_google” and “accordion.” We believe there lies the reason why the context information can solve the ambiguity coding problem. Further, to get more discriminative matrix, code words are reweighed as can be seen in Figures 3(b) and 3(d) (the details of reweighing can be seen in Section 4.1). After we repeat the above procedure over all classes, relationship matrixes for all classes are constructed. We denote it as {*H* ^1^, *H* ^2^ …, *H* ^nc^}, where nc is the number of classes.

We assume that every image of each class shares the common pattern in its local domain; thus, the relationship matrix that captures the partial pattern of image in local domain can be applied to describing the context information.

### 3.2. Context-Aware Locality-Constrained Linear Coding

After the context information has been described by the word-to-word cooccurrence matrix, it can be incorporated into the coding model. Let **X** be a set of *D*-dimensional local descriptors extracted from an image; that is, **X** = [*x*_1_,…, *x*_*N*_] ∈ **R** ^*D*×*N*^. Given a codebook with *K* entries, **B** = [*b*_1_, *b*_2_,…, *b*_*K*_] ∈ **R** ^*D*×*K*^, and relationship matrixes for all classes, [*H*_1_; *H*_2_ …; *H*_nc_]. Then, we incorporate this context information into coding step by solving the following problem with respect to the template matrix *H* ^*p*^ of *p*th class:

(9) ci=argminc||wxi−Bc||22+λ||α·dfi+1β·dci+1⊙c||22s.t.  1Tcw=1,

where **c**_*i*_ is the code for **x**_*i*_ and **c**_*ij*_ is the *j*th element of **c**_*i*_. **d**_*f*_*i*__ represents the distance between *x*_*i*_ and **B** in feature space the same as used in LLC [15]. **d**_*c*__*i*_ indicates the connected weight between *x*_*i*_ and **B**, which can be considered as the inverse distance between *x*_*i*_ and **B** in context space. Particularly,

(10) dci=exp⁡(conn(xi,B)σ),

where conn(*x*_*i*_, *B*) = [conn(*x*_*i*_, *b*_1_),…, conn(*x*_*i*_, *b*_*K*_)]^*T*^ and conn(*x*_*i*_, *b*_*j*_) represents the connected weight between *x*_*i*_ and *b*_*j*_, which is obtained from the template matrix *H* ^*p*^ according to the label of feature *x*_*i*_. And *σ* is used for adjusting the weight decay speed for the locality adaptor in context space. *λ* is the regularization parameter controlling the degree of constraint in feature space and context space, *α* indicates the weight of locality in feature space, and *β* indicates the weight of locality in context space. Indeed, parameters *α* and *β* can be controlled by parameter *λ*. The reason we introduce these two parameters is to compare the influence of **d**_*f*_*i*__ and **d**_*c*__*i*_ to model performance in experiment stage. If the label of *x*_*i*_ is *l*, then conn(*x*_*i*_, *b*_*j*_) is approximately calculated as follows:

(11) conn(xi,bj)=Hljp (l:the  label  of  xi).

The greater the value of *H*_*lj*_^*p*^ is, the closer the relationship between *x*_*i*_ and *b*_*j*_ is represented in class *p* and the shorter the context distance between *x*_*i*_ and *b*_*j*_ will be because *dc*_*i*_ represents their inverse context distance, and vice versa. As a result, the response coefficient of the corresponding code word *b*_*j*_ is greater. Therefore, from (9), we can see that the distance between *x*_*i*_ and **B** in both feature space and context space controlled the response of the code words simultaneously. Thus, those similar features with different context can be encoded discriminatively.

The factor *w* measures the similarity (context matching degree) between the cooccurrence relationship within the context domain of the feature being coded and the corresponding cooccurrence relationship from the template matrix *H* ^*p*^. The details of its calculation procedure are presented as follows. If *x*_*i*_ is the feature to be coded with the label of *l*, to calculate the parameter *w*, firstly, we find the features 〈*f*_*i*1_, *f*_*i*2_,…, *f*_*in*_〉 within the context domain of *x*_*i*_ and their corresponding label 〈*l*_*i*1_, *l*_*i*2_,…, *l*_*in*_〉. Then, for each feature in the context domain, we find the value of template matrix *H* ^*p*^ at (*l*, *l*_*ij*_), *j* = 1,…, *n*. Because the value in matrix *H* ^*p*^ represents the strength of the cooccurrence between two code words, the sum of those values with respect to all features can denote the degree of the centering feature fitting its context for the *p*th image category. Therefore, the corresponding *w* of *x*_*i*_ over *H* ^*p*^ can be calculated as

(12) wip=∑j=1nHp(l,lij)n.

Then, *w*_*i*_^*p*^ is regularized to [0 1] by using *w*_*i*_^*p*^ = exp⁡(2(*w*_*i*_^*p*^ − max⁡_*w*)), where max⁡_*w* is the max value of *w*_*i*_^*p*^  (*p* = 1,…, nc). Obviously, if *x*_*i*_ is extracted from the image in the *p*th category, the value of *w*_*i*_^*p*^ has a higher probability to be great because its local context is similar to the context of *p*th image category. Otherwise, the value of *w*_*i*_^*p*^ will be very small due to their dissimilar context. Additionally, from the analytical solution (details can be seen in the appendix) of (9), we can get the conclusion that the greater the value of *w*_*i*_^*p*^ is, the greater the value of coding coefficient is, and vice versa. Therefore, those noisy features will produce coding coefficient with small value because their context often does not match any template context, and they will be discarded in the pooling stage if we use the max pooling strategy to get the final signature. The details of above procedures are summarized in Algorithm 1.

For each *H* ^*p*^, *p* = 1,…, nc, we encode *x*_*i*_ by the above-presented method; then, we get the coding coefficient [*c*_*i*_^1^,…, *c*_*i*_^nc^], where *c*_*i*_^*m*^ denotes the coding coefficient corresponding to the relationship matrix *H* ^*m*^ for the *m*th image class. Therefore, given an image *I* with *N* descriptors **X** = {*x*_*i*_ ∈ *R* ^*D*^, *i* ∈ 1,…, *N*}, their coding coefficient matrix *c* = [*c*_1_^1^,…, *c*_1_^nc^, *c*_2_^1^,…, *c*_2_^nc^,…, *c*_*N*_^1^,…, *c*_*N*_^nc^] with respect to the relationship matrixes {*H* ^1^, *H* ^2^,…, *H* ^nc^} over all classes is obtained. Then, we obtain the final signature by max pooling [11] over matrix *c*, which is widely used in pattern recognition tasks [16, 17, 20] because it has been proven to be consistent with the properties of the cells in visual cortex [20]. Owning to the function of parameter *w*, the final signature mainly preserves the coding coefficient value over the class to which the feature really belongs.

## 4. Implementation 

In this section, we present the main details of word-to-word cooccurrence matrix construction, coding coefficient solving, and codebook learning due to their significant influence on the proposed model.

### 4.1. Discrimination of Word-to-Word Cooccurrence Matrix

The word-to-word cooccurrence matrix plays a key role in our method. As presented in Section 1, the reason why the ambiguity coding problem can be solved lies in that the context of ambiguous features often appears different. Therefore, more attention must be paid to the discrimination property of the relationship matrix. Intuitively, the more discriminative the word-to-word cooccurrence matrixes are, the better the performance of the model is. However, in realistic image, there are often many similar local appearances that exist in every class. For example, in the outdoor scenes, the sky often occupies very large space of the images. As a result, those features extracted from that space will be similar in terms of appearance and context, which degrades the discrimination of the relationship matrixes. As can be seen in Figures 3(a) and 3(c), some columns of the map are very light, which indicates that the corresponding code word appears close to all other words, resulting in that those code words are selected preferentially to encode any feature. To enhance the discrimination of the relationship matrix, the code word reweighing method is adopted. As demonstrated in [21], the purity of each code word is correlated with its discriminative power. To measure the purity of each code word quantitatively, we choose to use the entropy of each visual word's distribution in relationship matrix. The larger the entropy is, the less pure the code word and the smaller weight the code word should be, and vice versa. Let {*H* ^1^, *H* ^2^,…, *H* ^nc^} be the relationship matrixes over all classes, and the words relations distribution over all classes is calculated as

(13) HD=∑i=1ncHi.

Therefore, the relation distribution of the *i*th word is the *i*th column of *HD*. Let *e*_*i*_ represent the entropy of the *i*th word *b*_*i*_; then, *e*_*i*_ can be calculated as

(14) ei=−∑j=1ncHDjiln⁡(HDji).

The weight of the *b*_*i*_ can be calculated as

(15) wwi=exp⁡(−ei).

By using this reweighing method, the word (e.g., the lightest column of the map in Figures 3(a) and 3(c)) with large entropy will be reweighed to near zero. As a result, the discrimination of the relationship matrixes is enhanced. The effectiveness of this method can be seen in Figures 3(b) and 3(d).

### 4.2. Efficiency of Coefficient Solving

Unlike some coding strategies (e.g., SC, LSC, and LCSR) that need computational iteration to get the optimal coding coefficient, CALC has an analytical solution because its object function is convex. From (9), the analytical solution of CALC can be derived by

(16) ci=w(1TΨ1)−1(Ψ)−11,

where Ψ = 2(*Q* + *λ*diag⁡(**d**_*i*_)^2^), *Q* = (*x*_*i*_1^*T*^ − **B**)^*T*^(*x*_*i*_1^*T*^ − **B**), and

(17) di=α·dfi+1β·dci+1.

The details of its derivation procedure are in the appendix at the end of this paper. In implementation, to guarantee the low reconstruction error and computational complexity, we adopt the similar approximation strategy as used in [15]. Firstly, we select *k*  (*k* ≪ *K*) nearest basis of *x*_*i*_ in feature space as candidate basis in advance, and then *x*_*i*_ is encoded over these *k* bases using the proposed model. Indeed, this strategy forms a smaller codebook $\tilde{B}$ with the size of *D* × *k*, and then features are coded over it, which further improve the coding speed to a real-time level because the size of $\tilde{B}$ is much smaller than **B**. For a 255 × 255 image with 31 × 31 descriptors extracted, less than 0.5 second to solve their coefficient using a CPU with a frequency of 2.7 GHz is only spent.

### 4.3. Optimization of Codebook Training

In Section 3, we assume the codebook is given. A simple way to generate codebook is to use clustering based methods such as *k*-means [8]. As demonstrated in [15], the codebook generated by this kind of method is not optimal because clustering based method is a common approach and it does not consider the specific criteria (e.g., feature space locality and context space locality) of the current model. In this section, we train a more optimal codebook and analyze the algorithm of constructing codebook in detail. According to the codebook learning method presented in [18], the specific codebook learning model for CALC can be rewritten as

(18) min⁡C,B ∑i=1N(||xi−Bci||22+λ||α·dfi+1β·dci+1⊙ci||22)s.t. 1Tci=1, ∀i.

It must be noted that this codebook optimization formulation is different from the formulation in LLC [15]. The original LLC imposes the norm-bounded constraint |*b*_*i*_ | |≤1 in its codebook learning formulation, while in (18), this constraint is dropped. As demonstrated in [18], the benefits of dropping the norm-bounded constraint in (18) are twofold. First, we are able to obtain a codebook **B** which better fits the local data structure and favors classification. Second, closed-form solutions can be derived for both codebook update and sparse coding stages when solving (18), and thus faster convergence can be expected.

As suggested in [18], it can be solved using block coordinate descent or nonlinear Gauss-Seidel methods [22] to iteratively optimize **C**(**B**) based on existing **B**(**C**). We adopt the same steps of the codebook training method as in literature [18]. In the sparse coding stage (when **B** is fixed), the analytical solution of **C** exists and is unique as derived in (16). As for the codebook update stage (when **C** is fixed), we have the closed-form solution for **B** by setting its partial derivatives of *F*(**B**) to zeroes (see the details in [18]). Theoretically, such an iterative procedure will converge to stationary points [22]. The details of optimization procedure are presented in Algorithm 2, where **X**_*s*_ is the data randomly selected from the whole data, the initial codebook *B*_init_ is the average of each cluster, and the stopping criterion is that the objective function in (18) is no longer decreasing.

## 5. Analysis of CALC

*Evolution*. It is noted that this coding scheme degenerates into two particular cases when controlling the parameters *w*, *α*, and *β*. (1) When *w* = 1 and *α* = 1, *β* = 0, it just considers the locality in feature space and CALC degenerates into the case of LLC scheme. (2) When *w* = 1 and *α* = 0, *β* = 1, it degenerates into the case just considering the locality in the context space.

*Advantages*. Compared with the previous works such as VQ, SVQ, SC, LSC, and LLC, some advantages of CALC coding scheme can be presented here.

**Figure 4:**
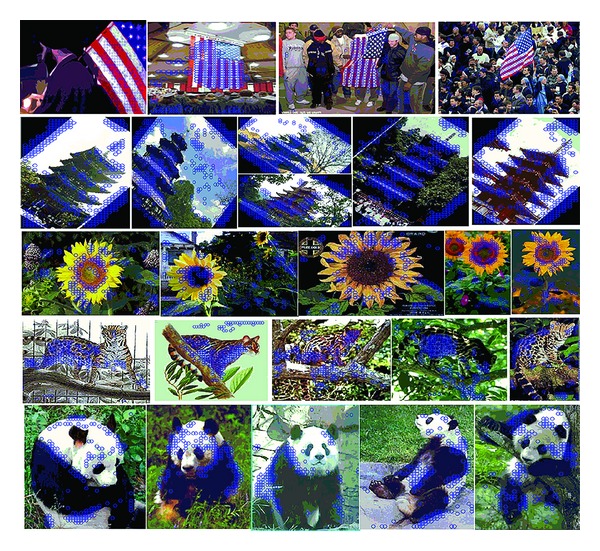
The noise removing functions of *w*. We select some categories from Caltech101 and Caltech256 for experiment. Local descriptors are densely extracted with step size of 8 pixels. And the blue circles represent the descriptors preserved under condition *w* > 0.4. As can be seen, most of the background descriptors are removed while the common features of each class are preserved. You may magnify this figure for details.

1. *Avoiding Coding Ambiguity.* CALC encodes the feature locally in both feature space and context space. The locality in feature space guarantees the reconstruction precision while in context space guarantees the semantic coding. Thus, coding ambiguity problem is originally handled in this paper by incorporating the context information into coding procedure; meanwhile, the reconstruction precision is also guaranteed.
2. *Noise Removing Ability.* In every image, there are lots of descriptors (e.g., the descriptors extracted from the clustering background in American flag as shown in first row of Figure 4) that are not only nonsense but also harmful for describing the image; we regard such descriptors as noise. By introducing the parameter *w* into CALC, the coefficient of noise is very small because its context does not often match any template context (corresponding *w* is small). As a result, the noise will not make any contribution to the final signature of the image with the max pooling operation. Its noise removing ability is also demonstrated by our experiment. And the experiment result can be seen in Figure 4.
3. *Fast Computational Speed Prospects.* Due to the convexity of its object function, CALC inherits the unique advantage, an analytical solution for the object function, from the LLC coding strategy. Furthermore, unlike LSC that encodes all features simultaneously when considering their relationship, CALC encodes those features independently while preserving their relationship. These advantages lead to a real-time speed prospect under the MapReduce framework [23] in cloud computing even though dealing with massive amount of images, which make significant sense for its realistic application.

Additionally, it must be noted that CALC is different from LCSR in terms of using context information. Although context information has been used in LCSR, our method is different from LCSR in the following two aspects: (1) the main motivation of considering the context information is different. In LCSR, coding consistency in terms of local spatial domain is their purpose, while, in this paper, we aim at making the coding semantically discriminative. (2) The context description style is also different. In LCSR, the similarity of spatially neighboring patches under a fixed neighboring system is measured to describe the context, while, in this work, a word-to-word cooccurrence matrix is learnt for every class.

## 6. Experiment and Analysis

In this section, we conduct experiments on three widely used image datasets Scene15 [19], Caltech101 [24], and Caltech256 [25] to evaluate the proposed method. On these datasets, with the common pipeline as adopted in [10, 12, 14, 16, 17, 20, 24], we evaluate the proposed method through the following aspects. First, the effectiveness of context consideration is evaluated by comparing with LLC because CALC is an enhancement of LLC. Second, the parameters' (including context size *r*, dictionary size *d*, and parameters *α*, *β*, *w*) selection of the proposed method is analyzed on Caltech101. Third, we compare the performance of CALC with the state of the arts on all three datasets.

### 6.1. Experiment Setting

Unless indicated otherwise, in all the experiments we conducted, common experiment setting is adopted as follows to ensure consistency. For all datasets, images are first resized to keep the maximum size of height and width no more than 300 pixels. Dense SIFT features [7] are extracted from all datasets from a single scale of 16 × 16 patches with the step size of 8 pixels. Fairly, codebooks, using both the method in Algorithm 2 and *k*-means [8], are trained on a randomly selected subset of SIFTs (~10^5^ SIFTs) belonging to the training dataset. The relationship matrixes are learned using 30, 60, and 100 images randomly selected from each category of Caltech101, Caltech256, and Scene15, respectively. The candidate basis size *k* is set to 10. For obtaining the final signature of the images, the max pooling [11] method is adopted and the SPM [19] strategy with three levels (1 × 1,2 × 2,4 × 4) is used. The linear SVM package [26] is used for the classification task because it showed good performance when combining with the max pooling method [11]. Following the standard experimental setting, we use randomly selected 30, 30, and 100 images per class for training while leaving the remaining for test on datasets Caltech101, Caltech256, and Scene15, respectively. All the experiments are conducted under 10 times repetition, and the average accuracy of each class is finally reported.

### 6.2. Datasets

*Scene15*. This dataset contains 4485 images fallen into 15 scene categories; the number of images of each class varies from 200 to 400. Scenes are captured from the environments varying from indoor to outdoor.

*Caltech101*. This dataset has 101 object categories and one background category, each containing from 31 to 800 images. In contrast to the two previous datasets, containing scene images, the current task rather deals with object recognition.

*Caltech256*. Caltech256 contains 29,780 images belonging to 256 categories besides a background class in which none of the images belongs to these 256 categories. Comparing with Caltech101 in which objects are often in the center of images, the intraclass variances in Caltech256 are much bigger (including object location and viewpoint). As a result, object recognition task is very challenging in this dataset.

### 6.3. The Effectiveness of Considering Context

**Figure 5:**
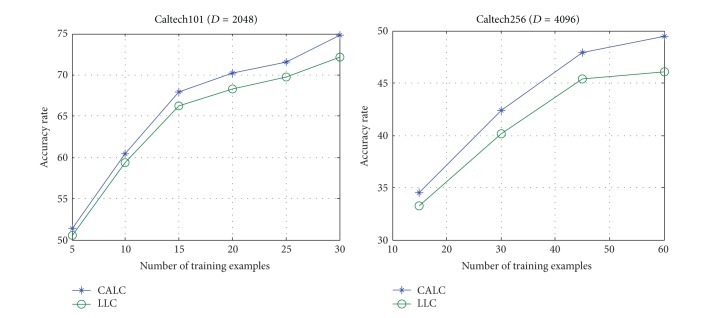
Comparison between CALC and LLC under different training examples.

To evaluate the effectiveness of context factor, we compare our method with LLC by reimplementing LLC [15] based on the codes provided by its authors. This comparison experiment is conducted on Caltech101, Caltech256 with such different settings: different number of training images per category under the two different coding schemes. For fair comparison, the codebooks are constructed by using *k*-means. In this experiment, we select the optimal parameters (*α* = 0.6, *β* = 0.4, *r* = 17) for CALC as analyzed in Section 6.4. As can be seen in Figure 5, our CALC outperforms LLC regardless of the size of the training images on Caltech101 and Caltech256. Hence, the effectiveness of considering context factor is demonstrated.

**Figure 6:**
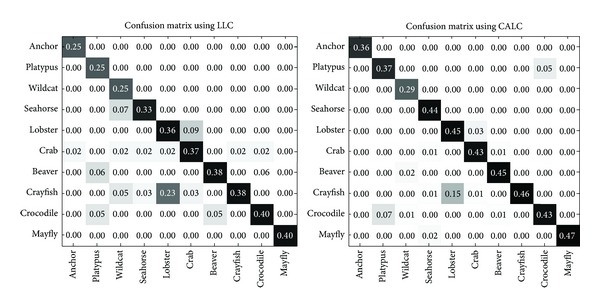
The details comparison of top 10 misclassified objects. This subconfusion matrix is extracted from the whole confusion matrix with size of 102 × 102.

Furthermore, we analyze the detailed classification rate improvement on top 10 misclassified categories in Caltech101 when using LLC. From Figure 6, we see that the classification rate of our method improves significantly (the highest improvements achieve 12% on “anchor” and “platypus”) to LLC on the majority of the categories even on those confused categories such as “lobster,” “crab,” and “crayfish.” We believe that this significant improvement may be due to the context consideration. These confused categories are similar in details, and they are easy to be misclassified by using LLC because it has no ability to solve the ambiguity problem, while by using CALC, this misclassification will be alleviated because the coding ambiguity is solved by considering context.

### 6.4. Parameter Analysis

On Caltech101, we studied the influence of the parameters *α*, *β*, and *w* and the size of local context domain *r* on our algorithm. For comparison, we restricted *α* + *β* = 1. The codebook in this experiment is learned by Algorithm 2 as presented in Section 4.1. In this experiment, two versions of *w*  (*w* = 1, *w* = *w*) are evaluated due to its significant importance to CALC, where *w* = *w* means that the value of *w* is calculated using (12) in Section 3.

**Figure 7:**
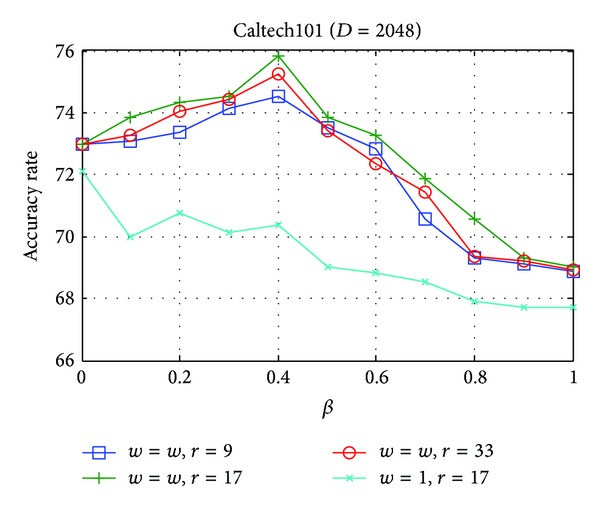
Performance of CALC under different parameters. We set *α* + *β* = 1 in this experiment.

As can be seen in Figure 7, when *w* = *w*, the main trend of the performance increases first and decreases then with the increase of *β* (with the descending of *α*). It implies that locality in both feature space and context space is important to the performance of coding. As presented, locality in feature space guarantees the reconstruction precision while in context space guarantees the semantic coding. The reason why the performance decreases as *β* further increases after achieving the top point may lie in that reconstruction error is not guaranteed when discarding the locality in feature space. It is worth noting that the best performance (75.84%) of CALC with this version improves the best result (74.9%) in Figure 4 about 1%, which is due to their difference styles in codebook construction; the former uses *k*-means and the latter uses Algorithm 2.

When *w* = 1, the performance of the CALC decreases with the increase of *β*. Note that in this case CALC degenerates into LLC when *β* = 0, and the performance of CALC with this version is even worse than LLC. This implies that *w* is very important to the performance of CALC and only with it the context consideration can be effective. We believe this may be because the max pooling in CALC is conducted over the coding coefficients corresponding to all the relationship matrixes, which leads to a confusion signature of image when *w* = 1. As a result, the performance is degraded. It also worth noting that when *β* = 0, the version of *w* = *w* outperforms the version of *w* = 1. Indeed, when *β* = 0, *w* = *w*, the CALC enhanced the LLC (*β* = 0, *w* = 1) through introducing a noise removing parameter *w*. This result also demonstrated the noise removing function of *w*.

Analytically, the context size *r* is also very important to the CALC model. When it is too small (e.g., *r* is smaller than the step size of feature extraction), the relationship matrix will be a diagonal matrix, which means there is no relationship among code words but self-to-self relationship. On the contrary, when it is too great (e.g., *r* is greater than the size of image), each row of the relationship matrix will be the statistic histogram of the whole image; thus, all rows are the same. These two extreme cases do not satisfy the discrimination property of relationship matrix, so *r* must be set to a balanced value so as to satisfy the discrimination property. On Caltech101, its optimized value is 17 as shown in Figure 7. Generally, as an empiric from the experiments on different datasets, it can be set to a value able to contain 2~3 neighboring features.

**Figure 8:**
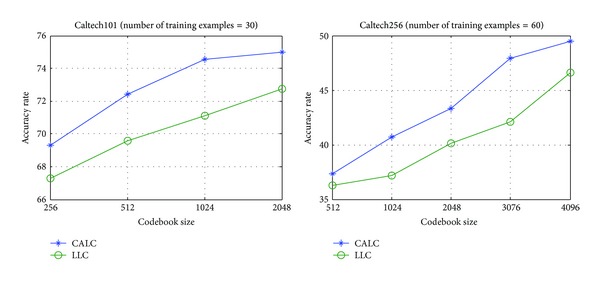
Performance comparison of CALC and LLC under different codebook sizes.

The influence of codebook size on proposed model is also studied. The codebook in this experiment is constructed using *K*-means. As can be seen in Figure 8, the CALC outperforms LLC regardless of the codebook size on Caltech101 and Caltech256. Additionally, with the increase of codebook size, the accuracy rate of our method improves slightly when the codebook size is enough (e.g., greater than 1024 on Caltech101), which is different from LLC in which the performance is sensitive to the codebook size. We believe this may be due to that, in LLC, the bigger the codebook is, the high probability the similar feature is encoded discriminatively, while in CALC, a small codebook is enough to encode the similar feature discriminatively due to its context consideration. As a result, with a smaller codebook size, our method achieves a comparable result with LLC using a far more bigger codebook size (e.g., similar performance with codebook size 512 on CALC and 2048 on LLC on Caltech101).

### 6.5. Comparison with the State of the Arts

In this subsection, we compare our method with several published methods on three datasets. Our comparison mainly focuses on the following two strategies: LSVQ and LLC, because these schemes are representatives of the state of the arts. We have to mention that the results of those schemes in the literatures [14, 15] are produced under different settings. For instance, LLC extracts multiscale feature every 8 pixels, a mix-order max-pooling operation is applied by LSVQ, the size of codebook varies from each other, and so forth. For fair comparison, we reimplement those methods using the same setting with our method. Meanwhile, comparing with other implementations provides a reference to evaluate the performance of our method. In this experiment, according to the parameter analysis result in Section 6.4, the same parameter setting *α* = 0.6, *β* = 0.4, *r* = 17 is adopted on all datasets. Following the setup of LLC, we train the dictionary with sizes of 1024, 2048, and 4096 on datasets Scene15, Caltech101, and Caltech256, respectively, using *k*-means. Additionally, to evaluate the effectiveness of dictionary optimization algorithm, the performance of CALC using dictionary learned by Algorithm 2 is also presented. As can be seen in Table 2, our algorithm outperforms majority of the methods on three datasets under our experiment setting. The performance utilizing learned dictionary improves *K*-means about 1%; therefore, the algorithm proposed in Algorithm 2 is effective. In detail, the accuracy rate of our implementation version of LLC is slightly worse than the published result in [15]. We think it may be due to the difference in feature extraction. Single scale level is adopted in our implementation while three scales are used in original LLC. It also must be noted that our implementation version of LSVQ achieves a higher accuracy than the original version on Scene15; the reason may lie in that our version's codebook size is larger than that of the original version. Additionally, our method outperforms all of the methods listed on Caltech101 and Caltech256 while performs not so perfectly on Scene15. In terms of this aspect, our method is more suitable for object recognition rather than scenes image classification. We believe this may be due to the fact that object often shares more similar context than scenes image within local domain. It also can be seen that the accuracy rate of our approach is far lower than LSVQ on Scene15. Nevertheless, on this dataset, our method is more computationally fast and it improves LLC and obtains comparable accuracy rate with the most of the listed methods.

## 7. Conclusion

To alleviate the ambiguity problem in coding, a novel improvement version of BOF named CALC with employing the context information is introduced in this paper. Since the context information describes the objects on the whole view, the proposed coding approach helps to alleviate the ambiguity problem and make the coding semantic at some degree. Furthermore, by introducing the feature weight parameter into the novel coding model, CALC has the ability to overcome the distortion problem produced by noisy feature. Experiment on several common used datasets demonstrated the effectiveness of the proposed method. Compared with the traditional strategies, this approach outperforms majority of the published methods in both Caltech101 and Caltech256. Furthermore, it inherits the unique advantage, an analytical solution, of LLC model, which leads to a real application prospect of this method. The experiment results also show that this method is more suitable for object recognition than scenes classification owning to the fact that object shares more common pattern in local domain than scenes share. Our future works will focus on the following aspects: (1) seeking a more robust context description method for both object and scenes image; (2) applying context information to object tracking task; and (3) conducting extensive experiment on other datasets.

## Figures and Tables

**Algorithm 1:**
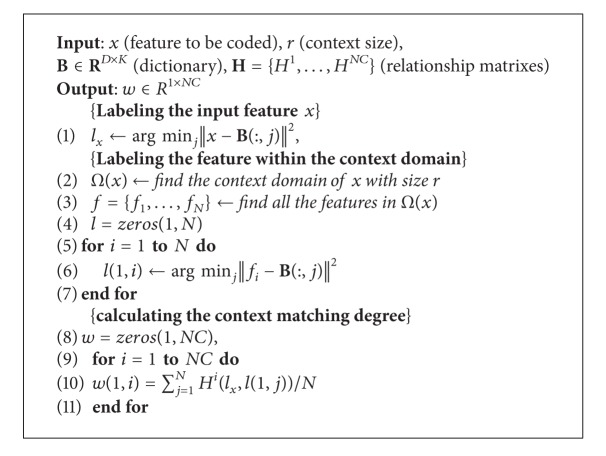
Context matching degree calculation.

**Algorithm 2:**
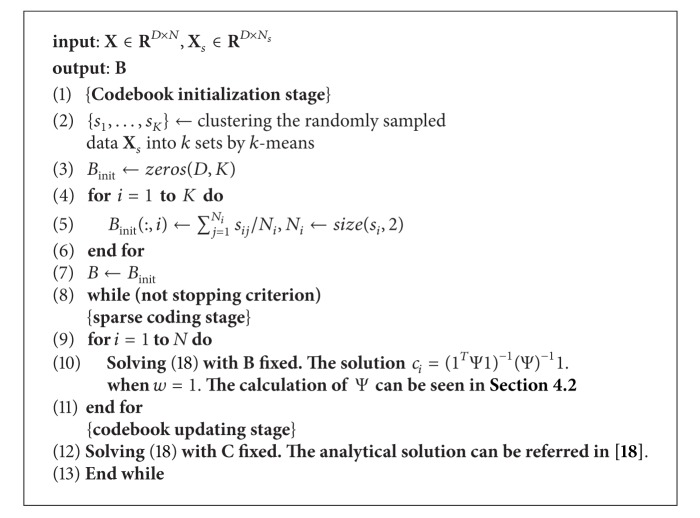
Codebook optimization.

**Table 1:** Comparison of previous coding schemes.

Coding scheme	Quantization error	Nonconsistent coding	Computational cost	Ambiguity
VQ [19]	High	Low	Low	High
SVQ [13]	Low	Low	Middle	High
SC [11]	Low	High	High	High
LSC [16]	Low	Low	High	High
LLC [15]	Low	Low	Low	High
LCSR [17]	Low	Low	High	High

**Table 2:** The comparison result with several coding styles on Caltech101 (training examples with size of 30), Caltech256 (training examples with sizes of 30, 60), and Scene15 (training examples with size of 100). Up the bold line are the results from the corresponding literature; below the line are the results from our implementation. And the two versions of CALC implemented are dictionary trained using *k*-means and Algorithm 2.

Unit: %	Cal. 101 (# 30)	Cal. 256 (# 30)	Cal. 256 (# 60)	Scene15 (# 100)
VQ [19]	64.60 ± 0.80	NA	NA	81.40 ± 0.50
SC [11]	73.20 ± 0.54	34.02 ± 0.35	40.14 ± 0.91	80.28 ± 0.93
LSC [16]	NA	35.74 ± 0.10	40.32 ± 0.32	**89.78** ± **0.40**
LLC [15]	73.44 ± NA	**41.19** ± **NA**	**47.68** ± **NA**	NA
LSVQ [14]	**74.21** ± **0.81**	NA	NA	82.70 ± 0.39
LCSR [17]	73.23 ± 0.81	NA	NA	87.23 ± 1.14

LLC [ours]	72.32 ± 0.91	40.32 ± 0.26	46.56 ± 0.78	81.73 ± 0.75
LSVQ [ours]	72.58 ± 0.79	38.51 ± 0.42	43.10 ± 0.11	**83.08 ± 0.56**
CALC (*k*-means)	**74.90** ± **0.44**	**42.37** ± **0.38**	**49.45** ± **0.67**	81.89 ± 0.54
CALC (learned)	**75.84** ± **0.56**	**43.12** ± **0.62**	**51.44** ± **0.92**	82.53 ± 0.81

## Appendix

### The Derivation Procedure of (16)

To determine the solution of *c*_*i*_, we consider the Lagrange function *L*(*c*_*i*_, *η*), which is defined as

(A.1) L(ci,η)=||wxi−Bci||22+λ||α·dfi+1β·dci+1⊙ci||22+η(1Tciw−1).

Denote (*α* · **d**_*f*__*i*_ + 1)/(*β* · **d**_*c*__*i*_ + 1) as **d**_*i*_ and considering the constraint 1^*T*^ *c*_*i*_/*w* = 1, the above formula can be derived as

(A.2) L(ci,η)=||(wxi1Tw−B)ci||22+λ||di⊙ci||22+η(1Tciw−1),

which can be reformed as

(A.3) L(ci,η)=ciTQci+λciTdiag⁡(di)2ci+η(1Tciw−1),

where *Q* = (*x*_*i*_1^*T*^ − **B**)^*T*^(*x*_*i*_1^*T*^ − **B**), diag⁡(**d**_*i*_) is a diagonal matrix whose nonzero elements are the entries of **d**_*i*_.

Let ∂*L*(*c*_*i*_, *η*)/∂*c*_*i*_ = 0; we have

(A.4) Ψci+ηw1=0,

where Ψ = 2(*Q* + *λ*diag⁡(**d**_*i*_)^2^). Once we premultiply (A.4) by 1^*T*^Ψ^−1^, we obtain

(A.5) 1TΨ−1Ψci+1TΨ−1ηw1  =1Tci+1TΨ−1ηw1  =w+1TΨ−1ηw1=0.

So *η* = −*w* ^2^(1^*T*^Ψ^−1^1)^−1^; submitting *n* into (A.4) gives the analytical solution

(A.6) ci=w(1TΨ1)−1(Ψ)−11.
